# Chemical Composition, Antioxidant Activity, and Antibacterial Activity of Black Poplar Buds’ Hydroalcoholic Macerates from Dobrogea Area

**DOI:** 10.3390/molecules28134920

**Published:** 2023-06-22

**Authors:** Gabriela Stanciu, Florin Aonofriesei, Simona Lupsor, Elena Oancea, Magdalena Mititelu

**Affiliations:** 1 Department of Chemistry and Chemical Engineering, 4 Mamaia Blvd., Ovidius University of Constanta, 900527 Constanta, Romania; gstanciu@univ-ovidius.ro; 2 Department of Natural Sciences, Faculty of Natural and Agricultural Sciences 1, Ovidius University of Constanta, University Street, 900527 Constanta, Romania; 3 S.C. Dan-Elis SRL, Product Manufacturing, 9 Dobrogei Street, 907285 Topraisar, Romania; oancea.careless@gmail.com; 4 Clinical Laboratory and Food Hygiene Department, Faculty of Pharmacy, ”Carol Davila” University of Medicine and Pharmacy, 3-6 Traian Vuia Street, 020956 Bucharest, Romania; magdalena.mititelu@umfcd.ro

**Keywords:** black poplar buds’ macerate, phenolic compounds, DPPH radical scavenging test, HPLC, metallic concentration, phytocompounds

## Abstract

Black poplar buds have high contents of many compounds with therapeutic potential, which are useful in cosmetics and the treatment of various dermatitis, respiratory diseases, etc. The aim of this study was to identify and exploit the local plant resources with biologically active properties from the Dobrogea area, Romania. For this purpose, materials were collected from the mentioned area, and macerates of black poplar were prepared in order to evaluate their qualities as antioxidant and antimicrobial agents. Three different black poplar buds’ hydroalcoholic macerates were analyzed by the Folin–Ciocâlteau method to estimate the total content of phenolic compounds, by the HPLC-DAD method for identification and quantification of the main bioactive compounds and by the DPPH radical scavenging method to evaluate the antioxidant activity. All hydroalcoholic macerates showed high concentrations of phenolic compounds, the main individual compounds being gallic acid, chlorogenic acid, cinnamic acid, and methyl gallic acid. The antioxidant activity of the black poplar buds’ hydroalcoholic macerates, evaluated by the DPPH radical scavenging test, showed high values, between 496 and 1200 mg GAE /100 g d.w. The Cd, Cu, Zn, Ni, and Pb concentrations released in dry poplar buds, determined by AAS, were below the detection limits. Hydroalcoholic macerates of black poplar were tested against two groups of gram-positive bacteria (*Enterococcus* and *Staphylococcus*) using an agar well diffusion assay. The in vitro inhibitory activities of the macerates were important and ranged from 8.2–9.4 mm inhibition zones (*Staphylococcus)* to 8.6 −10 mm inhibition zones (*Enterococcus*).

## 1. Introduction

Poplars (*Populus* spp.) are widely spread all over the world, including more than one hundred species distributed in temperate and subtropical regions [1,2]. Species of *Populus* have been used in folk medicine, specifically for their anti-inflammatory properties [3]. Among the *Populus* species, the black poplar (*Populus nigra*) is widely distributed in Europe.

In recent years, nutraceuticals have attracted growing attention because of consumers’ increasing concerns about their health, which has facilitated intensive research in this field [4]. Various extracts of poplar leaves and buds are used as flavor sources, especially in in alcoholic beverages, in many commercially available tea formulas and in several other forms (balsam, poplar oil, tincture, etc.) in traditional medicine. Many of these products are used empirically, without any phytochemical and biological characterization.

The Populus species has great potential for therapeutic applications: in the treatment of dermatitis, rheumatism, upper respiratory tract infections, and skin aging [5,6,7,8,9]. Poplar bud extract has also been reported for use in the treatment of oropharyngeal cavity, urinary tract, digestive/excretory tract, and bacterial infections [3,7,10]. Exploring the antimicrobial properties of natural products has become a significant research effort [11,12,13,14,15]. A significant antimicrobial effect of the biological components present in the buds, leaves, and branches of black poplar has been reported [14]. The resinous exudate of black poplar (*Populus nigra*) constitutes the basic element of European propolis and, in general, of that of temperate zones [16,17].

It was found that the resinous exudate of the black poplar (*Populus nigra*) buds contained the following: the flavonoid aglycons; the flavanones pinocembrin and pinostrobin; the flavonols galangin, quercetin, and kaempferol; the flavones chrysin and apigenin; and the esters of phenolic acids [8,16]. It has been reported that poplar buds’ extracts contain phenolic glycosides (salicin and populin), phenols (rosmarinic acid, caffeic acid, ferulic acid, quercetin, chlorogenic acid, and p-coumaric acid) [16], volatile oil (sesquiterpenes, sesquiterpenoids, and derivates of benzoic acid) [18,19], tannins, resins, gallic acid, saponins, and flavonoids (chrysin, tectochrysin, galangin, pinocembrine, naringenin, and taxifolin). The content of salicylates and flavonoids from poplar extracts are mainly responsible for their anti-inflammatory, analgesic, spasmolytic, and antibacterial properties [20]. *Populus nigra* L. buds have been described mainly to contain phenolic compounds, flavonoids, and terpenoids (more than 150 substances) [8]; therefore, a lot of liquid and gas chromatographic techniques have been developed to determine all chemical compounds, particularly GC- MS [21,22,23] and HPLC [24,25,26,27,28].

It is important to consider that plants, in general, are also an important source of bio compounds, and particularly, it is important to consider that poplars are used to extract heavy metals from soil and water. Therefore, additional data regarding the metallic composition of the analyzed samples before extraction are always important.

The chemical composition varies depending on the species, the geographical region, and the time of sampling [29,30,31]. Significant data have revealed that the chemical composition of buds of the Populus species is directly influenced by the types of Populus species, geographical growth, and climate [32,33]. Poplar species, which are used by bees as a source of propolis in Europe, have a similar qualitative composition of bud exudates but can be very different in quantitative composition [14].

The black poplar is very widespread in the Dobrogea region, from the Black Sea coast to the Danube Meadow, in the plains and the hilly area. It is an unpretentious species, resistant to drought and frost. It is used ornamentally in gardens and parks.

Considering the data from the specialized literature, this study aims to capitalize on these natural resources for cosmetic purposes. In order to establish the optimal formula for extracting the active principles from black poplar buds, three types of solvents were tested: ethanol 97%—sample P3, ethanol–distilled water 6.5:2 (*v*/*v*)—sample P2, and ethanol–silver-fir water 6.5:2 (*v*/*v*)—sample P1 (original composition).

The obtained hydroalcoholic macerates of black poplar buds were characterized by determining the total content of phenolic compounds and the antioxidant capacity; the antibacterial potential was tested against two groups of gram-positive bacteria (*Enterococcus* and *Staphylococcus*) by an agar well diffusion assay. The mineral composition of the dried buds was also analyzed to determine the possible degree of toxicity.

## 2. Results and Discussion

### 2.1. Total Phenolic Content (TPC)

The content in polyphenolic compounds of the obtained hydroalcoholic macerates, determined by the Folin–Ciocâlteau method, shows high values between 1387.5–1989.25 mg/100 g d.w., in the ascending order P2 < P3 < P1 (third column of Table 1. This corroborates that the most efficient extraction solvent is silver-fir water, the original solvent described for the first time in this work (Section 3.4). Silver-fir water can have a synergistic effect on the extraction of phenolic compounds existing in black poplar buds or can contribute with its own compounds to increase the concentration of total phenolic content in the P1 macerate. Our previous research on the composition of some plants used in cosmetics corroborated that the aqueous distillates show antioxidant activity, mainly due to the content of polyphenolic compounds [34].

According to data from the literature, the extraction solvents can significantly influence the profile of the separated polyphenolic compounds. Finding the optimal solvent for extracting biologically active compounds from plants is a very topical problem [8].

Our previous research corroborates that the most suitable solvents for the extraction of antioxidant compounds are the hydroalcoholic ones.

In order to establish the identity of the main separated phenolic compounds, present in the three macerates, the use of the HPLC-DAD technique was required.

### 2.2. Phenolic-Compound Separation, Identification, and Quantification

Hydroalcoholic macerates analyzed by HPLC showed high total phenolic-compound concentrations, between 989–1814 mg/100 g d.w. (Table 2).

In sample P1, only four phenolic compounds (chlorogenic, gallic, cinnamic, and ellagic acids) were identified under the analytic conditions used. Gallic and chlorogenic acids showed high concentrations above 660 mg/100 g d.w., ellagic acid having the lowest concentration. Sample P2 contained eight of the nine determined phenols; cinnamic and chlorogenic acids predominated with values of approximately 350 mg/100 g d.w. In sample P3, cinnamic and methyl gallic acids were the main compounds, with values of approximately 515 and 350 mg/100 g d.w., respectively.

Among the analyzed compounds, cinnamic acid was found in significant concentration in all three samples. Caffeic acid, reported in the literature as being in large quantities in black poplar buds, was found in small quantities only in samples P2 and P3.

The identity of the polyphenolic compounds from the black poplar buds differs significantly in relation to the geographical area of origin and therefore to the geoclimatic conditions. Thus, in Serbia, caffeic and p-coumaric acids predominate, and in Italy, France, and the United Kingdom, caffeic and isoferulic acids, with coumaric and cinnamic acids being in low concentrations [8,19].

Gallic acid, a natural botanic phenolic compound, which can mediate various therapeutic properties that are involved in anti-inflammation, anti-obesity and anti-cancer activities [35], has been found in all three studied macerates, sample P1 having the highest concentration. Methyl gallate, a gallic acid derived compound, has been recently found responsible for the inhibition of hepatic cell carcinoma proliferation [36]. Sample P3 shows the highest methyl gallic acid concentration.

**Figure 1:**
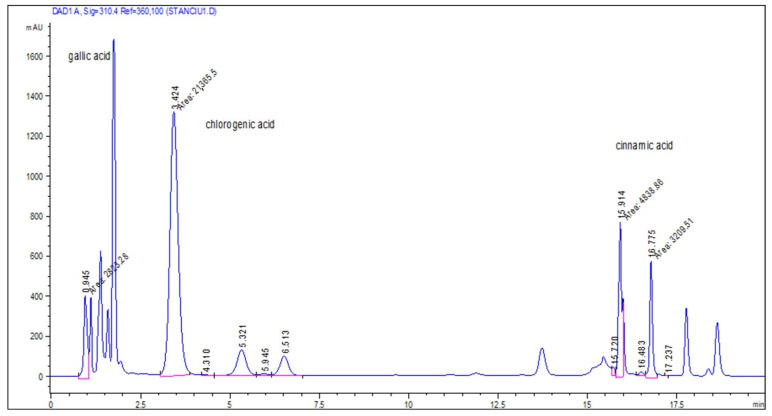
The sample P1’s chromatogram.

Chlorogenic acid (CGA), found in a higher concentration in the P1 sample (Figure 1), is able to exert pivotal roles on glucose and lipid metabolism regulation and on the related disorders, e.g., diabetes, cardiovascular disease (CVD), obesity, cancer, and hepatic steatosis. Potential health benefits of CGA include anti-diabetic, anti-carcinogenic, anti-inflammatory, and anti-obesity impacts [37].

Therapeutic potential of cinnamic acid and its derivatives includes anti-inflammatory, antioxidant, antimetastatic, photoprotective, anti-diabetic, anti-melanogenesis, neuroprotective, antiviral, antimalarial, antituberculosis, antibacterial, antifungal, and antiparasitic [8,38,39]. All three studied samples registered good values of cinnamic acid concentrations, but the highest was in the P3 sample.

Ellagic acid, found in all analyzed macerates, has a lot of therapeutic properties: anti-inflammatory, antioxidant, antimetastatic, vasoprotective, cardioprotective, anti-diabetic, neuroprotective, hepatoprotective, photoprotective, anti-obesity, antihypertensive, anti-melanogenesis, antibacterial, antiviral, and antituberculosis [8].

Table 1 shows the correlation between the results of TPC and HPLC determinations. The TPC values of samples P1, P2, and P3, obtained by the Folin–Ciocâlteau method (Table 1, third column) are comparable to the total phenolic compounds determined by HPLC (Table 1). It can be seen that macerate P1 has a higher concentration, followed by P3, and P2 has the lowest value. Table 1 presents also the percentage of the total phenolic compounds measured by HPLC from TPC determined by the Folin–Ciocâlteau method, as well as the main phenolic compounds determined as previously discussed. Sample P1 shows the best match between the compared techniques, having 91.18% of individual phenols measured by HPLC versus TPC.

### 2.3. DPPH Radical Scavenging Test

The hydroalcoholic macerates studied have a highly complex composition of bioactive compounds with beneficial effects on the skin, making them difficult to analyze and identify within a short period of time. Assessing the antioxidant capacity of plant extracts for cosmetic use using the DPPH test is a simple and effective method [34]. The antioxidant capacity is mainly due to the content of phenolic compounds but also of other compounds, such as minerals and vitamins.

The antioxidant activity of black poplar buds’ hydroalcoholic macerates, evaluated by the DPPH radical scavenging test, showed high values, between 496 and 1200 mg GAE /100 g d.w. (Table 3), being in perfect concordance with previous measurements (TPC and HPLC). Sample P1, with the highest content of total polyphenolic compounds, has the highest antioxidant capacity, and sample P2, with a significantly lower content of polyphenolic compounds, has an antioxidant capacity several times lower. The macerate obtained with ethanol and silver-fir water (sample P1) showed the highest phenolic-compound concentration and antioxidant activity, which could be explained by synergic effect.

### 2.4. Metallic Concentration Measurement

The analysis of the mineral composition of black poplar buds is necessary to demonstrate the opportunity of safety in using them for cosmetic purposes.

Metallic concentrations have been measured in poplar buds, after proper mineralization using flame atomic absorption spectrometry. Table 4 presents the correlation coefficients of calibration curves and the mean values of metallic concentrations, measured in triplicate. Major (Ca, Mg, and Na) and minor (Fe and Mn) elements registered significant values of concentration, while toxic-elemental (Cd, Cu, Ni, Pb, Zn) concentrations were below the detection limits.

Calcium is an essential element for all higher plants, but plants differ greatly in the amounts of calcium they need. Calcium pectate is an important component of plant cell walls. Of all the analyzed metals, calcium recorded the highest concentrations, with 5538.00 mg/kg d.w. in black poplar buds’ dry product. Magnesium is a component of the chlorophyll molecule, essential in the process of photosynthesis, and plants cannot do without it. Although not essential for most plants, sodium can be beneficial to plants under many conditions, especially when potassium is deficient. As such, it can be considered a non-essential or functional nutrient. A concentration of 523.70 mg/kg d.w. of sodium has been found in the dried product of black poplar buds. Iron, as an essential element for all plants, has many important biological roles, participating in numerous enzymatic reactions and plant respiration. Iron and manganese play an important role in plant growth and development but often compete for absorption, as an abundance of one of these micronutrients makes the other less available to plant roots.

These results confirm the possibility of using black poplar buds from the Dobrogea area for cosmetic or therapeutic purposes.

### 2.5. Agar Well Diffusion Evaluation of Antibacterial Activity in Black Poplar Extracts

Recorded values showed good antibacterial potential (Table 5 and Table 6). On average, the lowest activity was observed for sample P3 (8.3 to 8.6 mm inhibition zones). The maximal inhibitory power (10 mm) showed sample P2 against *Enterococcus* strains, while *Staphylococcus* seemed to be more sensitive to sample P1 (Table 6).

Overall, *Enterococcus* showed to be a little more sensitive to black poplar macerates than *Staphylococcus*. Our results are in agreement with other reports dealing with bioactive components found in different species of poplar. In a study carried out on the effect of propolis macerates obtained from poplar by Widelski et al., 2022 [40], it was noted that they had a very good antibacterial activity with the minimum inhibitory concentration (MIC) fluctuating between 1.25 and 5.00 mg/mL compared to most gram-negative bacteria tested. Also, the activity of the macerates was particularly promising against *Helicobacter pylori*, its growth being inhibited by concentrations of 0.03 mg/mL of poplar macerate. Promising results were also obtained against gram-positive bacteria (0.08 and 0.31 mg/mL) and three species of *Candida* (0.31 mg/mL) [40]. Another study showed that poplar bud extracts have variable antibacterial activity that depends on the type of plant material (fresh or dried) as well as the extraction method (infusion, maceration, decoction, or ultrasound) [41]. These authors found that, in general, the ethanolic macerates from dry material have higher antibacterial activity, and it was manifested especially against gram-positive bacteria. The inhibition zones found by the authors for *S. aureus* (17.3–18.7 mm) and *E. faecalis* (16.7–19.3 mm) were consistently larger and most likely determined by the chemical composition of macerates.

Poplar bioactive components can inhibit microorganisms isolated from infections [14], especially gram-positive bacteria [42]. The components with antimicrobial properties are often concentrated on poplar bud exudates [15]. Some reports showed that poplar extracts can act synergistically enhance the action of some antibiotics [43]. Antimicrobial activity of poplar components depends on the species, geographic region, and season [29,30,44,45,46,47]; therefore, reports on their antimicrobial activity may vary according to the source. The inhibitory activity also seemed to vary depending on the poplar species used for the macerates. Thus, it was observed that the inhibitory activity against *S. aureus* and *Candida albicans* was approximately twice as strong when the macerates were made from black poplar buds compared to balsam poplar extracts [48].

Presented data indicate promising potential of black poplar components in controlling bacterial growth, potential that may find practical applications in a variety of fields.

## 3. Materials and Methods

### 3.1. Plant Materials

*Populus nigra* L. buds were collected in June 2022 from an organic culture in Topraisar, Constanta County, Romania.

The poplar buds were dried at ambient temperature until constant weight was achieved and grinded to obtain a powder of a proper degree of fineness. Powder plant material was subjected to maceration.

### 3.2. Chemicals

All used chemical agents for chemical determinations were of analytical reagent grade.

Gallic acid was purchased from Fluka (Buchs, Switzerland) and the Folin–Ciocâlteau reagent from Merck (Darmstadt, Germany). The solution of gallic acid (standard phenolic compound) of 1 × 10^−2^ mol × L^−1^ was prepared by dissolving 0.1881 g of gallic acid in 100 mL of methanol/H_2_O 1:1 (V:V). The Folin–Ciocâlteau reagent was diluted with distilled water 1:2 (V:V).

DPPH (2,2-difenil-1-picrililhidrazil) was purchased from Aldrich (Hamburg, Germany). The standard compound solution of 0.063% (1.268 mM) was prepared in a 200 mL calibrated flask by dissolving 0.1000 g of 2,2-difenil-1-picrililhidrazil in methanol.

### 3.3. Apparatus

The chromatographic determinations of phenolic compounds were performed with the HPLC-DAD system Agilent 1200. Molecular absorption spectrometric measurements were carried out using a UV-VIS JASCO V550 scanning spectrophotometer. Metallic concentrations were determined by atomic absorption spectrometry with an AA 700 spectrometer provided by Analytic Jenna, Jena, Germany.

### 3.4. Sample Preparation

The selection of solvents was based on the results of previous research and also on observations from cosmetic practice [12,13,26,34,43,45]. Ethanol is a commonly used solvent in herbal extractions due to its ability to dissolve a wide range of biologically active compounds. The results of previous studies indicated that hydroalcoholic solvents may be more effective for the extraction of a wider range of polyphenolic compounds.

To obtain the studied hydroalcoholic macerates, ethanol 97% prepared by fermentation of grains was used. Table 7 presents the details of samples P1, P2, and P3 preparation.

The distillate (2 L of silver-fir water with pH 4.02) was kept 40 days until it reached pH 3.5. At this point, the silver-fir water was ready to use to obtain macerate P1.

All hydroalcoholic macerates were vacuum filtrated and analyzed.

The metallic determination was done by the AAS method. A quantity of 0.5 g of dry poplar buds’ powder was mineralized with 5 mL of nitric acid and 40 mL of deionized water to 120 °C for 130 min, filtered in 50 mL volumetric flasks and filled up with deionized water.

### 3.5. Identification and Quantification of Phenolic Compounds by HPLC-DAD

The obtained hydroalcoholic macerates were analyzed by HPLC-DAD using the adapted USP30 HPLC method [13,43] for identification and quantification of the phenolic compounds.

Separation was carried out on the Zorbax Eclipse XDB-C18 column (250 mm, 4,6 mm; 5 µm) (Agilent Technologies). The gradient elution was done using phosphoric acid of 0.1% in water (solvent A) and acetonitrile (solvent B), as presented in Table 8.

The chromatographic working parameters were 1.5 mL/min for the flow rate, 20 µL injection volume, and 22 min analysis time.

Quantification of the phenolic acid was done using absorbance measurements at 310 nm and 35 °C. The retention times and DAD spectra were compared to available authentic standards. A mixture of standard solutions in 70% methanol was used, having the following concentrations: 37 mg/mL of E—resveratrol, 0.22 mg/L Z—resveratrol, 0.36 mg/mL caffeic acid, 0.37 mg/mL chlorogenic acid, 0.58 mg/mL cinnamic acid, 0.42 mg/mL vanillin, 0.39 mg/mL gallic acid, 0.48 mg/mL ferulic acid, 0.34 mg/mL 3-methylgalic acid, 0.43 mg/mL ellagic acid, and 0.51 mg/mL p-coumaric acid.

The retention times of standard solutions were determined (Table 9). Standard deviations of retention times were obtained after statistical processing of the six injections (soft SPSS 10).

Identification and quantitative determination of the active constituents from samples’ extractive solution were done by comparing the samples’ chromatograms with the chromatogram of the standard mixture.

### 3.6. Total Phenolic Content (TPC) Analysis

The total phenolic contents were determined according to the Folin–Ciocâlteau method using gallic acid as the standard [26,44]. The absorbance was measured at 681 nm. The total phenolic content of the poplar buds’ hydroalcoholic macerates was expressed in mg of gallic acid equivalents per 100 g of dry weight (mg GAE/100 g d.w.). All samples were measured in triplicate, and the mean value was reported.

#### Calibration Curve

The volumes of 1 mL, 2 mL, 3 mL, 4 mL, 5 mL, 6 mL, and 7 mL of gallic acid standard solution were introduced in 50 mL volumetric flasks each and after was treated with 1 mL of the Folin–Ciocâlteau reagent 1:10 (V:V) and 1 mL of 20% (*w*/*v*) aqueous Na_2_CO_3_; after 10 min, the volume was brought to mark with distilled water. All samples were incubated for 30 min at 25 °C, and the absorbance was measured at 681 nm.

The calibration curve was linear in the range of 0.68–4.76 mg GAE/L, the equation being y = 0.1082x−0.0511, with a correlation coefficient of 0.9994. The total phenolic content of poplar buds’ macerates was expressed in mg of gallic acid equivalents per 100 g of dry weight (mg GAE/100 g d.w.). All samples were measured in triplicate, and the mean value was reported.

For TPC analysis, volumes of 10 mL of each sample (P1, P2, and P3) were transferred into a 50 mL volumetric flask and brought to the mark with the same solvent previously used for the maceration operation.

To measure the total phenolic content, volumes of 1 mL of previously diluted samples were added in 50 mL calibrated flasks each, then treated with 1 mL of the Folin–Ciocâlteau reagent 1:2 (V:V) and 1 mL of sodium carbonate solution at 20%, and then, the process was the same as for calibration.

### 3.7. DPPH Radical Scavenging Test

The antioxidant capacity was evaluated using the DPPH radical scavenging test [45,46]. Gallic acid (GA) was used as the standard to plot the calibration curve, and the results were expressed as equivalents (mg GAE). In 25 mL calibrated flasks, different volumes of 0.5 mL, 1.0 mL, 1.5 mL, 2.0 mL, 2.5 mL, and 3.0 mL of gallic acid standard solutions were added; then, 5 mL of DPPH at 0.063% (1.268 mM) in methanol was added. They were filled up to the mark with methanol and left in the dark to reach room temperature for 45 min before the absorbance registration was performed at 530 nm versus methanol. Previously, the DPPH solution spectrum was recorded, and the maximum absorbance was registered at 530 nm.

The calibration curve with gallic acid as the standard was linear in the 0.54–2.72 mg GAE/L range, and the correlation coefficient was 0.9973.

To measure the antioxidant capacity, 1 mL of each sample was added in 25 mL calibrated flasks; then, 5 mL of DPPH at 1.268 mM in methanol was added. They were filled up to the mark with methanol and left in the dark to reach room temperature for 45 min before the absorbance registration was performed at 530 nm using methanol as the blank.

### 3.8. Agar Well Diffusion Evaluation of Antibacterial Activity of Black Poplar Extracts

Black poplar hydroalcoholic macerates (P1, P2, and P3) were tested against 25 gram-positive bacterial strains (belonging to the genera *Enterococcus* and *Staphylococcus*) (Table 10).

The potential antibacterial activity of black poplar extracts was estimated using an agar well diffusion assay [44,45,49]. Overnight cultures of the tested bacterial strains were inoculated in Mueller–Hinton broth and incubated for 20 h at 37 °C. Cultures were then diluted with physiological sterile water (8.5 g/L NaCl) to reach a bacterial density between 8 × 10^5^ and 1 × 10^6^ UFC/mL. Inoculations were made by flooding the Mueller–Hinton agar surface with 1 mL of bacterial suspension. The excess of inoculum was then removed, and media were left to dry for 1 h. Wells were cut aseptically into Mueller–Hinton agar, and every well received 150 µL of macerate. After diffusion of macerates, inoculated media were placed into the incubator (for 48 h at 37 °C). The effect of macerates was estimated as inhibition zones measured from the edge of wells. Triplicate determinations were performed for each sample. Results are expressed as mean ± SD (standard deviation) of triplicate analysis.

## 4. Conclusions

Black poplar buds represent an accessible and important source of valuable compounds for maintaining health in general and, in particular, for cosmetic use. Considering the proposed goal, the conducted research revealed the following:

1. The most efficient formula for extraction showed to be the mixture of ethanol–silver wire water 6.5:2 (*v*/*v*)—sample P1 (original composition). The macerate obtained with ethanol and silver-fir water (sample P1) showed the highest phenolic-compound concentration and antioxidant activity. This observation could potentially be explained by a synergistic effect between the ethanol-and-silver-fir-water components.
2. The present study identified and highlighted the compounds responsible for antioxidant activity in various hydroalcoholic macerates of black poplar buds from the Dobrogea region. These compounds include cinnamic acid, chlorogenic acid, gallic acid, and methyl gallic acid. Among the analyzed compounds, cinnamic acid was found in significant concentration in all three samples.
3. The antioxidant activity of hydroalcoholic macerates of black poplar buds was assessed using the DPPH radical scavenging test. The results showed high values, ranging from 496 to 1200 mg GAE/100 g d.w. These findings are consistent with previous measurements of total phenolic content (TPC) and high-performance liquid chromatography (HPLC), demonstrating a strong correlation between antioxidant activity and the presence of phenolic compounds in the macerates.
4. The results of toxic-metallic concentration are below the limit of detection, confirming the possibility of using black poplar buds from the Dobrogea area for cosmetic or therapeutic purposes. The ranking for metallic concentrations in black poplar buds is Ca > Na > Mg > Fe > Mn.
5. The antibacterial activity was significant against *Staphylococcus* and *Enterococcus* and can be appreciated as an important property of black poplar buds’ macerates. This feature could be exploited in a variety of practical applications as an adjuvant in controlling the growth of pathogenic or opportunistic bacteria.

## Figures and Tables

**Table 1:** Correlation between the results regarding the total concentration of phenols in black poplar hydroalcoholic macerates obtained by molecular spectrometry and HPLC-DAD.

No.	Sample	TPC mg GAE/100 g d.w.	Percent of Total Measured Individual Phenols by HPLC from TPC, wt. %	The Major Individual Determined Phenols by HPLC	
				Phenolic Compound	wt.%
**1**	**P1**	**1989.25**	91.180	Chlorogenic acid	33.305
				Gallic acid	34.474
2	P2	1387.5	71.282	Chlorogenic acid	24.600
				Cinnamic acid	26.281
3	P3	1872.5	68.78	Cinnamic acid	17.938
				Methyl gallic acid	12.272

d.w. = dry weight.

**Table 2:** Contents (mg/100 g d.w.) and weight percentages (wt.%) of individual phenolic compounds of tested *Populus nigra* hydroalcoholic macerates determined by HPLC-DAD.

Phenolic Compound	P1		P2		P3	
	mg/ 100 g d.w.	wt.%	mg/ 100 g d.w.	wt.%	mg/ 100 g d.w.	wt.%
Chlorogenic acid	662.536	36.527	341.325	34.511	1.829	0.142
Caffeic acid	-	-	9.123	0.922	71.661	5.563
Gallic acid	685.773	37.809	138.006	13.954	128.335	9.963
Methyl gallic acid	-	-	21.665	2.191	352.531	27.369
Cinnamic acid	375.699	20.714	364.65	36.869	515.280	40.005
Vanillin	-	-	0.285	0.029	-	-
Ferulic acid	-	-	4.383	0.443	-	-
Ellagic acid	89.79	4.950	109.600	11.081	190.329	14.777
*p*-Coumaric acid	-	-	-	-	28.090	2.180
**Total phenolic content**	**1813.798**	**100**	**989.037**	**100**	**1288.055**	**100**

d.w. = dry weight.

**Table 3:** The antioxidant activity of black poplar buds’ macerates.

No.	Sample	DPPH mg GAE/100 g d.w.
1	P1	1200.12
2	P2	496.875
3	P3	1057.5

d.w. = dry weight.

**Table 4:** Metallic concentrations (mg/kg d.w.) in black poplar buds’ dry product.

Metal	Concentration (mg/kg d.w.) ± SD	Correlation Coefficient of Calibration Curve
Ca	5538.00 ± 1.15	0.9997
Fe	75.92 ± 2.04	0.9992
Mg	494.60 ± 2.12	0.9963
Mn	17.20 ± 1.42	0.9968
Na	523.70 ± 3.33	0.9956
Cd	<DL	0.9956
Cu	<DL	0.9991
Ni	<DL	0.9948
Pb	<DL	0.9950
Zn	<DL	0.9954

SD—standard deviation, DL—detection limit, d.w. = dry weight.

**Table 5:** Inhibitory activity of black poplar macerates on *Enterococcus* strains (mm inhibition zone) (mean ± SD).

Bacterial Strain	Sample		
	P1	P2	P3
*Enterococcus 1*	9 ± 0.57	9 ± 0.28	10 ± 0.50
*Enterococcus 2*	10 ± 0.18	10 ± 0.33	10 ± 0.66
*Enterococcus 3*	10 ± 0.26	10 ± 0.55	9 ± 0.25
*Enterococcus 4*	12 ± 0.48	10 ± 0.66	9 ± 0.16
*Enterococcus 5*	10 ± 0.21	10 ± 0.12	8 ± 0.27
*Enterococcus 6*	12 ± 0.38	12 ± 0.24	8 ± 0.13
*Enterococcus 7*	9 ± 0.15	9 ± 0.22	7 ± 0.33
*Enterococcus 8*	12 ± 0.18	12 ± 0.62	8 ± 0.66
*Enterococcus 9*	9 ± 0.28	8 ± 0.11	8 ± 0.19
*Enterococcus 10*	9 ± 0.36	10 ± 0.34	9 ± 0.20
**Average activity**	**9**	**10**	**8.6**

SD—Standard deviation.

**Table 6:** Inhibitory activity of black poplar macerates on *Staphylococcus* strains (mm inhibition zone) (mean ± SD).

Bacterial Strain	Sample		
	P1	P2	P3
*Staphylococcus 1*	10 ± 0.28	9 ± 0.66	10 ± 0.52
*Staphylococcus 2*	12 ± 0.14	10 ± 0.22	9 ± 0.21
*Staphylococcus 3*	10 ± 0.33	8 ± 0.39	7 ± 0.42
*Staphylococcus 4*	10 ± 0.45	10 ± 0.51	9 ± 0.57
*Staphylococcus 5*	9 ± 0.32	9 ± 0.24	7± 0.61
*Staphylococcus 6*	9 ± 0.26	9 ± 0.55	7 ± 0.72
*Staphylococcus 7*	9 ± 0.15	8 ± 0.12	8 ± 0.13
*Staphylococcus 8*	9 ± 0.27	9 ± 0.28	7 ± 0.59
*Staphylococcus 9*	8 ± 0.54	8 ± 0.17	9 ± 0.34
*Staphylococcus 10*	9 ± 0.68	9 ± 0.35	9 ± 0.21
*Staphylococcus 10*	8 ± 0.22	9 ± 0.48	8 ± 0.18
*Staphylococcus 12*	8 ± 0.11	10 ± 0.16	8 ± 0.32
*Staphylococcus 13*	10 ± 0.19	8 ± 0.33	8 ± 0.76
*Staphylococcus 14*	10 ± 0.25	9 ± 0.28	9 ± 0.63
*Staphylococcus 15*	10 ± 0.66	8 ± 0.15	8 ± 0.14
**Average activity**	**9.4**	**8.6**	**8.2**

SD—Standard deviation.

**Table 7:** The studied hydroalcoholic macerates preparation.

Description	Sample		
	P1	P2	P3
Dry buds powder mass (g)	100	100	100
Ethanol 97%, volume (mL)	200	200	300
Dilution solvent, volume (mL)	silver-fir water *, 650	distilled water, 650	-
Maceration time at room temperature	3 months	3 months	3 months

* The silver-fir water was prepared by distillation of a mixture of plant and water (1 kg of fresh silver-fir sprouts and leaves with 4 L of distilled water).

**Table 8:** The gradient of elution solvents.

Time (min)	Solvent A, %	Solvent B, %
0–13	90	10
13	78	22
13	78	22
14	60	40
17	60	40
17.5	90	10
22	90	10

**Table 9:** The retention times of standards.

Standard	Retention Time ± SD
gallic acid	0.990 ± 0.025
3-*o*-methyl gallic acid	2.606 ± 0.008
chlorogenic acid	3.501 ± 0.015
caffeic acid	4.598 ± 0.036
vanillin	6.919 ± 0.051
*p*-coumaric acid	7.187 ± 0.019
ferulic acid	8.565 ± 0.058
*E*—resveratrol	14.467 ± 0.017
ellagic acid	15.303 ± 0.027
*Z*—resveratrol	15.751 ± 0.058
cinnamic acid	15.867 ± 0.007

SD—standard deviation.

**Table 10:** Bacterial strains used to test the effect of black poplar buds’ hydroalcoholic macerates.

Bacterial Strain	Observation
*Enterococcus 1–10*	Isolated from urinary tract infection (UTI)
*Staphylococcus 1–10*	Isolated from skin infection (SI)
*Staphylococcus 11–15*	Isolated from upper respiratory infection (URI)

## Data Availability

Not applicable.

## Sample Availability

Samples of the compounds are available from the authors.
